# Irregular Periapical Radiopacity in Mandibular Premolars and Molars

**DOI:** 10.1155/2014/910843

**Published:** 2014-02-20

**Authors:** S. Aravind Warrier, Divya Vinayachandran

**Affiliations:** ^1^Department of Oral Medicine and Radiology, Faculty of Dental Sciences, Sri Ramachandra University, Chennai 600 116, Tamil Nadu, India; ^2^Department of Oral Medicine & Radiology, SRM Kattankulathur Dental College and Hospitals, Kattankulathur 603 203, Kancheepuram District, Tamil Nadu, India

## Abstract

Increased deposition of cementum is observed in a wide number of both benign and malignant conditions. Many cases are often diagnosed during routine examination as an incidental finding. Diagnosing correctly without confusing it with other similarly appearing lesions, thus avoiding subjecting the patient to unnecessary investigations and stress, is of prime importance. We report one such case, where the patient presented with the routine complaint of a painful tooth, during the investigation of which he was also diagnosed with hypercementosis affecting the mandibular second premolars and molars bilaterally. The literature review reveals that not many cases of hypercementosis are frequently reported.

## 1. Case Report

**Figure 1:**
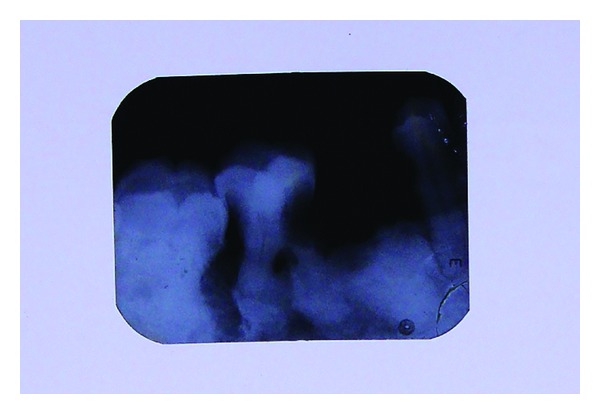
Intraoral periapical radiograph of the lower right mandibular region showing a coronal and radicular radiolucency involving the pulp of the right mandibular first molar and a root stump of the adjacent second premolar region. The roots of the second premolar and the first and the second molars were bulbous with an irregular outline.

**Figure 2:**
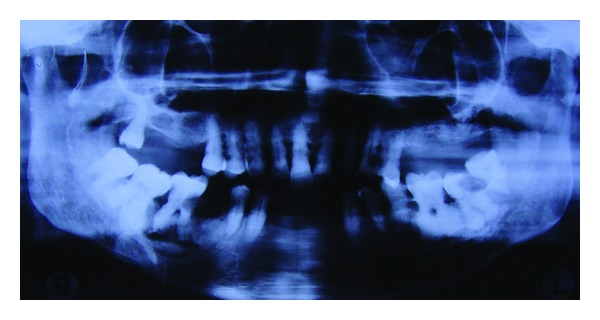
Extraoral imaging: conventional orthopantomogram confirming the presence of bulbous roots with irregular outline, bilaterally seen in the second premolars and first and second molars and revealing multiple missing teeth with generalized interdental bone loss.

**Figure 3:**
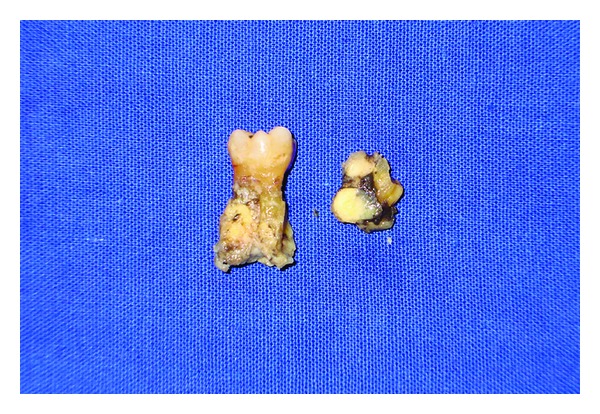
Specimen photograph taken following extraction of the carious first molar and the root stump of the second premolar, with irregular, yellowish calcified material on the root surface.

**Figure 4:**
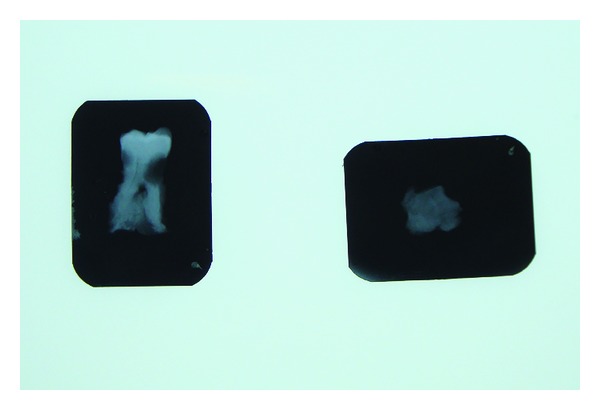
Specimen radiograph confirming the presence of a hard calcified radiopaque material on the root surfaces. A clear demarcation is visible between the primary root surface and the secondary deposition.

A 60-year-old male patient reported to a private outpatient clinic with the chief complaint of pain in the right lower back tooth region for a duration of two weeks. The patient's past medical history was uneventful and he was apparently healthy. On intraoral examination, root stump of the mandibular second premolar was seen, surrounded by inflamed gingiva, and the adjacent first molar was affected by caries, supraerupted and mobile. Both the teeth were associated with tenderness. An intraoral periapical radiograph of the region was taken (Figure 1). The radiograph confirmed the clinical findings of a deep carious lesion involving the pulp of the right mandibular first molar and a root stump in relation to the adjacent second premolar region. The radiograph also revealed an interesting appearance of the roots of the mandibular right second premolar and the first and the second molars. They appeared to be bulbous with an irregular outline. An orthopantomogram was taken, which revealed similar bulbous appearance of the roots of the left second premolar and the first and the second molars (Figure 2). The root stump and the first molar were surgically removed. The extracted root stump and the first molar revealed the presence of an additional yellowish, hard substance firmly attached to the root surface with an irregular outline (Figure 3). The specimen radiograph revealed an irregular radiopacity around the root (Figure 4). The original outline of the root was visible within the radiopaque mass. On histopathological examination the final diagnosis was hypercementosis. Patient was reviewed after one week and the healing was satisfactory.

## 2. Discussion

The term cementum is derived from the Latin word cementum or “cement,” meaning the stone particles used to make mortar, ideally describing its role [1]. The cementum covers the radicular portion of the tooth, providing attachment to the periodontal ligament fibers, for tooth articulation. Cementum is deposited by cementoblasts, in a layered manner, with each new layer being mineralized by the previous layer. Cementum deposited prior to the tooth eruption is referred to as primary cementum and that formed following eruption is the secondary cementum [1, 2]. Tooth cementum is a dynamic tissue which, in contrast to bone, does not undergo remodelling but rather is continuously deposited throughout the life of an individual, usually in response to functional demands.

Excessive deposition of cementum occurs in a wide spectrum of neoplastic and nonneoplastic conditions, such as benign cementoblastoma, cementifying fibroma, periapical cemental dysplasia, florid cementoosseous dysplasia, hypercementosis, and other benign fibroosseous lesions of periodontal ligament origin [3]. Hypercementosis is a nonneoplastic condition in which excessive secondary cementum is deposited. The literature review reveals that reports on hypercementosis are not several [2, 4].

Hypercementosis may be localized to a single tooth or few teeth in a quadrant or may be generalized affecting multiple teeth. The local causes suggested include an adaption to functional changes, trauma, inflammation [5]. Generalized hypercementosis is not very common and hereditary or systemic disturbances are considered to be etiological factors [5, 6]. Paget's disease, hyperthyroidism, rheumatic fever, rheumatoid arthritis, acromegaly, calcinosis, and vitamin A deficiency have all been implicated. Alternatively, an idiopathic, age related phenomenon has been considered as the most common cause. The early type seen in younger age groups is rare [7]. Basdra et al. (1997) reported one such case of hypercementosis in an 18-year-old female patient, with the need of orthodontic treatment. The patient had undergone orthodontic treatment previously. The patient's medical history was noncontributory. Radiographic examination revealed generalized hypercementosis affecting all mandibular teeth. The maxillary teeth were not affected. On comparing with the radiographs, previously taken during the earlier orthodontic management, in the mixed dentition stage, there was no presence of hypercementosis. Such cases of generalized hypercementosis, in the absence of any underlying systemic factors, at such an early age have not been frequently reported in the literature.

Extent and pattern of cementum deposition vary among tooth groups and tooth surfaces [6]. In a study of 22,000 patients approximately around 42 years of age, single-tooth hypercementosis was radiographically observed in 1.7% of the individuals. There is an increased predilection in the mandibular premolars and molars when compared to the maxilla [4, 6]. Bilateral involvement is also not uncommon. Cementum may be deposited in a generalized manner, causing a symmetric enlargement of the entire root, or may be deposited in a localized manner, leading to varied macroscopic appearances such as focal, circular, and club-shaped lesions [2, 8]. It has been commonly described as a tooth with a bulbous root [9]. An increased tendency to reduce root surface concavities has been observed leading to deposition of thicker cementum layers in root surface grooves and in the furcation of multirooted teeth [6]. Hypercementosis most commonly presents with no clinical signs and symptoms, although it may be associated occasionally with ankylosis when the cementum is in direct contact with the bone or with fusion of roots of adjacent teeth in certain circumstances [4, 6].

Radiographically, teeth affected by hypercementosis may show either a generalized or localized enlargement. Generalized hypercementosis is commonly either of the dense type or the transparent type [2]. In the dense type, the secondary cementum has the same density as the primary cementum and dentin. In the transparent type, the demarcation between the secondary and primary cementum and dentin can be clearly visualized. Occasionally, the secondary cementum formation is focal, having a bulbous or nodular appearing close to or at the root apex. The periodontal ligament and the lamina dura are always seen on the outer aspect of hypercementosis, surrounding it as in primary cementum [4, 9]. The biological width between the root surface, the periodontal membrane space, and the alveolar bone remains unaltered [9, 10].

Microscopically, hypercementosis appears as thick layers of cementum characterized by symmetric, highly basophilic lines parallel to the dentin surface [10].

Since hypercementosis presents with no clinical signs or symptoms, it is conservatively managed. Surgical excision is usually indicated in suspicious, atypical cases [4, 5].

## 3. Conclusion 

Hypercementosis is a rarely reported frequent condition. When presented with a case, it is important to differentiate it from other similarly appearing lesions and additionally rule out any underlying systemic causes. It is important to identify the need for invasive management. Additionally, such cases may present an endodontic or orthodontic challenge, which has to be managed accordingly.

## References

[1] Consolaro A, Consolaro RB, Francischone LA (2012). Cementum, apical morphology and hypercementosis: a probable adaptive response of the periodontal support tissues and potential orthodontic implications. *Dental Press Journal of Orthodontics*.

[2] Langland OE, Langlais RP, Preece JW (2002). *Principles of Dental Imaging*.

[3] Leider AS, Garbarino VE (1987). Generalized hypercementosis. *Oral Surgery, Oral Medicine, Oral Pathology*.

[4] Souza LN, Lima SM, Pimenta FJGS, Souza ACRA, Gomez RS (2004). Atypical hypercementosis versus cementoblastoma. *Dentomaxillofacial Radiology*.

[5] Stavrou E, Tosios KI, Stavrou IE (2007). Globular radiopacity around the apex of an impacted maxillary third molar. *Oral Surgery, Oral Medicine, Oral Pathology, Oral Radiology and Endodontology*.

[6] Suter VGA, Reichart PA, Bosshardt DD, Bornstein MM (2011). Atypical hard tissue formation around multiple teeth. *Oral Surgery, Oral Medicine, Oral Pathology, Oral Radiology and Endodontology*.

[7] Basdra EK, Stellzig A, Komposch G (1997). Generalized hypercementosis in a young female patient. *Oral Surgery, Oral Medicine, Oral Pathology, Oral Radiology, and Endodontics*.

[8] Zustin J, Friedrich RE (2010). Hypercementosis and odontogenic epithelial hyperplasia associated with a tooth root remnant mimicking a neoplasm. A case report. *In Vivo*.

[9] Manson-Hing LR (1959). X-ray evidence of mechanical trauma. *Oral Surgery, Oral Medicine, Oral Pathology*.

[10] Pinheiro BC, Pinheiro TN, Capelozza ALA, Consolaro A (2008). A scanning electron microscopic study of hypercementosis. *Journal of Applied Oral Science*.

